# “That pulled the rug out from under my feet!” – adverse experiences and altered emotion processing in patients with functional neurological symptoms compared to healthy comparison subjects

**DOI:** 10.1186/s12888-015-0514-x

**Published:** 2015-06-24

**Authors:** Astrid Steffen, Johanna Fiess, Roger Schmidt, Brigitte Rockstroh

**Affiliations:** 1 Department of Psychology, University of Konstanz, P.O.Box 905, Konstanz, 78457 Germany; 2 Neurological Rehabilitation Center Kliniken Schmieder, Eichhornstraße 68, Konstanz, 78464 Germany

**Keywords:** Functional neurological symptoms, Somatoform dissociation, Conversion, Dissociative movement and sensibility disorders, Adverse childhood experiences, Life events, Alexithymia, Suppression, Emotion regulation, Building block effect

## Abstract

**Background:**

Medically unexplained movement or sensibility disorders, recently defined in DSM-5 as functional neurological symptoms (FNS), are still insufficiently understood. Stress and trauma have been addressed as relevant factors in FNS genesis. Altered emotion processing has been discussed.

The present study screened different types and times of adverse experiences in childhood and adulthood in patients with FNS as well as in healthy individuals. The relationship between stress profile, aspects of emotion processing and symptom severity was examined, with the hypothesis that particularly emotional childhood adversities would have an impact on dysfunctional emotion processing as a mediator of FNS.

**Methods:**

Adverse childhood experiences (ACE), recent negative life events (LE), alexithymia, and emotion regulation style were assessed in 45 inpatients diagnosed with dissociative disorder expressing FNS, and in 45 healthy comparison subjects (HC).

**Results:**

Patients reported more severe FNS, more (particularly emotional) ACE, and more LE than HC. FNS severity varied with emotional ACE and negative LE, and LE partially mediated the relation between ACE and FNS. Alexithymia and suppressive emotion regulation style were stronger in patients than HC, and alexithymia varied with FNS severity. Structural equation modeling verified partial mediation of the relationship between emotional ACE and FNS by alexithymia.

**Conclusions:**

Early, emotional and accumulating stress show a substantial impact on FNS-associated emotion processing, influencing FNS. Understanding this complex interplay of stress, emotion processing and the severity of FNS is relevant not only for theoretical models, but, as a consequence also inform diagnostic and therapeutic adjustments.

**Electronic supplementary material:**

The online version of this article (doi:10.1186/s12888-015-0514-x) contains supplementary material, which is available to authorized users.

## Background

In contrast to psychoform dissociative symptoms such as dissociative amnesia, the understanding of dissociation experienced in the body (i.e., somatoform dissociative symptoms), still seems insufficient. Recently, the term functional neurological symptoms (FNS) has been introduced within DSM-5 to denote a heterogeneous group of medically unexplained neurological phenomena that can be discussed as a consequence of somatoform dissociation (e.g., [1, 2]).^1^

The labeling problem is only one sign of the manifold difficulties that arise when individuals show gait disturbances, do not feel parts of their body or are paretic, and when the neurologist cannot find a medical explanation. These patients might be referred to a psychiatrist or a psychotherapist, who is most likely to diagnose a dissociative or a conversion disorder (cf. ICD-10). Multiple professions will be involved in and challenged by their treatment [2].

The prevalence of FNS, their severity, and symptom-imposed psychological strain vary with an increasing risk of comorbid disorders [3] and seriously affect individuals’ quality of life. Still, an insufficient understanding of the nature and genesis of FNS may account for poor treatment outcomes [4–7].

“That pulled the rug out from under my feet!” – a common metaphor for losing stability when facing an adverse experience, is an expression noticeably often used by individuals with prominent motor FNS (e.g., gait disturbances). They use it  to describe their feelings during adverse experiences in the past. Stressful and traumatic experiences have been considered central to FNS generation ever since Freud’s and Janet’s models [8] (cf. [4]). Empirical studies have suggested specific types (such as sexual abuse or emotional neglect) and/or times of stress exposure (such as adverse childhood experiences, ACE, or recent life events, LE) as crucial factors for dissociative symptoms; that is, for *severe,* chronic dissociative symptoms [3, 4, 9–13]. For instance, the effects of frequent, extreme traumatic experiences on dissociations in patients with dissociative identity disorder (with the most extreme and enduring dissociative symptoms) are explained as the structural dissociation of psychological and biological systems and of emotional and apparently normal parts of the personality as a consequence to threatened personal integrity [8, 14–16]. Likewise, posttraumatic stress disorder (PTSD) has been associated with the process of shutdown-dissociation following extreme trauma-induced helplessness [17]. Hence, FNS have been reported in traumatized women diagnosed with PTSD [18] and in women with a significant degree of sexual traumata (e.g., [9, 12, 19]).

Regarding the time of experience, traumatic experiences in childhood during sensitive developmental periods were considered influential as they affect the development of neuroendocrine and brain structures and may thereby foster vulnerability to further stressful experiences (e.g., [20–23]). In line with this model, an impact of childhood emotional abuse on FNS has been reported in patients with a diagnosis of conversion disorder [24, 25]. Moreover, a relation between childhood experiences and critical adult life events has been reported for dissociative personality disorder (e.g., [16]), but also other severe mental disorders such as depression and borderline personality disorder [26]. Roelofs and colleagues [11] advocate for a multifactorial stress model involving a complex of early and later negative life events for FNS. Most studies focus on multiple, severe and chronic FNS, as present in patients diagnosed with conversion disorder (ICD-diagnosis F44.7) or dissociative identity disorder.

FNS has not only been associated with dissociations following extreme trauma [16, 17]: FNS patients have also reported high loads of emotional stress and abuse (e.g., [9, 10, 13, 27]). Moreover, patients with *somatoform* disorders [28] often present alexithymia, or the incapability to adequately perceive and verbally express emotions and feelings [29].

Thus, altered emotion processing and emotion regulation could be relevant factors in the development of FNS. Altered emotion processing can be conceived of as the redirection of emotional expressions into bodily expressions (i.e., conversion). The latter has been theoretically associated with alexithymia, but empirical evidence of this association is insufficient [29, 30]. Furthermore, altered emotion processing may be reflected in habitual tendencies to suppress emotional expressions together with impaired cognitive elaboration of emotional conflicts ([31], see also [28, 32–34]). Still, empirical evidence on emotion regulation in patients with FNS or dissociative disorder is insufficient, as is evidence on the interaction of both stress and emotion variables in a sample with FNS covering sensory and/or motor domains. Specifying the roles of stress and emotion processing in their relationship, however, should improve the understanding of FNS as a ‘conversion’ or dissociation disorder.

The present study addressed stress load and emotion processing as factors related to FNS generation by screening the time and type of adverse childhood experiences (ACE), recent life events (LE), alexithymia and emotion regulation styles in patients with ICD-10 diagnoses of a dissociative disorder with somatoform dissociative symptoms (i.e., FNS) from a single or multiple sensory or motor domains. A comparison with a sample of healthy subjects and regression analyses examined the hypotheses that: (1) FNS severity varies with the amount of adverse experiences, in particular emotional ACE and LE; (2) ACE predict later LE, and LE mediate the relationship between ACE, in particular emotional ACE and FNS; and (3) FNS severity varies with altered habitual emotion processing manifest in alexithymia and suppressive emotion regulation style, which both mediate the relationship between emotional ACE and FNS.

## Methods

### Participants

45 inpatients meeting diagnoses of dissociative disorder (ICD codes F44.4, F44.6, F44.7) were recruited from the local neurological rehabilitation center (Kliniken Schmieder Konstanz and Gailingen). Patients were diagnosed by at least two experienced psychiatrists and neurologists using ICD-10 guidelines. Diagnostic criteria included functional neurological symptoms (FNS^2^) with at least one core negative somatoform dissociative symptom, such as motor disorders or hypesthesia. Patients with so-called positive somatoform dissociative symptoms, such as dissociative seizures (ICD-code F44.5) and central nervous lesions (e.g., degenerative disorders, tumors) were excluded. 15 patients met criteria of dissociative motor disorder/dissociative movement disorder (ICD-code F44.4) or dissociative anaesthesia and sensory loss/dissociative sensitivity disorder (ICD-code F44.6), while 30 patients met the criteria of multiple dissociative movement and sensitivity disorders (ICD-code F44.7).

45 healthy comparison subjects (HC) were recruited by advertisement and flyer from the local community to be comparable in age and gender to the patient sample. Exclusion criteria were any sign of a current or lifetime mental disorder (screened with the German version of the M.I.N.I. International Neuropsychiatric Interview [35]) or neurological disorder as well as the use of psychoactive medication. Table 1 shows that groups did not differ in age and gender, while the HC group had more years of school education than patients.

**Table 1:** Sociodemographic information of study samples

	FNS patients			HC	FNS patients vs. HC
	Overall	F44.4/F44.6	F44.7		
*n*	45	15	30	45	
Gender (f/m)	32/13	11/4	21/9	31/14	Chi^2^ = .05, *p* = 0.82
Age (*M* ± *SD*)	40.4 ± 13.9	39.1 ± 16.1	41 ± 12.9	44.8 ± 15.3	*t*(88) = 1.44, *p* = 0.16
Years schooling (*M* ± *SD*)	10.1 ± 1.7	10.1 ± 1.6	10.1 ± 1.8	11.5 ± 1.6	*t*(88) = 3.9, *p* < 0.001
PTSD (*n*)	11	1	10	0	Chi^2^ = 12.53, *p* < 0.001

*Note.* FNS = functional neurological symptoms; F44.4/F44.6 = dissociative movement or sensitivity disorders; F44.7 = multiple dissociative movement and sensitivity disorders; HC = healthy comparison subjects; PTSD = posttraumatic stress disorder; f = female; m = male

### Procedure and assessments

The study protocol was approved by the ethics committee of the University of Konstanz as well as the board of the neurological rehabilitation center. Participants were informed about the goals and procedures of the study and gave their written informed consent prior to participation.

#### The severity of functional neurological symptoms

(FNS) was verified with the Somatoform Dissociation Questionnaire (SDQ-20 [36]; German Version by [37]). The SDQ-20 assesses the frequency (percentage) of somatoform dissociation experienced during the preceding twelve months.^3^ In addition to FNS symptom scores, general psychological strain (Symptom Checklist-90-R [38]; see [39]^4^) and comorbid diagnosis of posttraumatic stress disorder (PTSD; Posttraumatic Stress Scale-Interview, PSSI [40, 41]^5^) served to evaluate the severity of illness.

#### Stressful experiences

comprised adverse childhood experiences (ACE) and recent life events (LE). ACE were screened using the German version of the Early Trauma Inventory (ETI^6^ [42]; German version by [43]; see [44]). The ETI assesses the amount (number and frequency) of adverse childhood experiences before the individual onset of puberty^7^ in the four domains of general traumata, emotional abuse/neglect, physical abuse/neglect and sexual abuse. Negative and positive life events over the preceding twelve months were screened using the Life Events Questionnaire (LEQ^8^ [45, 46]).

#### Emotion-processing

indices were alexithymia and emotion regulation style. Alexithymia was assessed with the Toronto Alexithymia Scale (TAS-26^9^; [47–49]), habitual emotion regulation styles (suppression and cognitive reappraisal) were quantified in the Emotion Regulation Questionnaire (ERQ^10^ [31], German version [50]).

For detailed psychometric characteristics of each self-report instrument used, please see Additional file 1.

### Statistical analyses

An a priori G*Power software [51, 52] estimation of required sample sizes recommended a sample size of 44 participants to obtain sufficient effect sizes on linear multiple regression in a random model (one-tailed with three predictors and an error probability of α = 0.05, while power (1 - β) = 0.95 and ρ^2^ = 0.33), and on t-tests comparing two dependent means (one-tailed with α = 0.05, while power (1 - β) = 0.95 and the expected effect size dz = 0.5).

The impact of stressful experiences on FNS (hypothesis 1) was verified in two separate repeated-measures ANOVAs, both including group (FNS vs. HC) as a between-subject factor. In the first ANOVA, the within-subject factor ACE domain compared the four ETI domains (using the Greenhouse-Geisser epsilon correction); in the second ANOVA the within-subject factor LE compared positive and negative LEQ scores. The differences of emotion processing measures (TAS-26 and ERQs) and intensity of experienced psychological strain (SCL90R-GSI) between FNS and HC were examined using independent sample t-tests. Additional diagnostic subgroup comparisons of FNS patients with multiple (ICD code F44.7) or single symptom domains (ICD code F44.4 or F44.6) were analyzed for ACE, LE, the emotion processing measures and the intensity of psychological strain. These subgroup differences (as well as ANOVA post-hoc tests) were calculated with non-parametric Mann–Whitney U-tests reporting exact significance values, since assumptions for t-tests were not fulfilled. Chi square analyses were used to assess gender differences and the amount of comorbid PTSD diagnosis.

A forced-entry multiple regression analysis evaluated the contribution of the different variables (emotional ACE, negative LE, emotion processing measures) on symptom severity. Significant predictors of FNS were included in further calculations. A mediation analysis evaluated the impact of negative LE (hypothesis 2) and emotion processing measures (hypothesis 3) on the relationship between emotional ACE and FNS. Using a non-parametric resampling approach suggested by Preacher and Hayes [53], path coefficients were estimated in a multiple mediator model and bias-corrected bootstrap confidence intervals for both total and specific indirect effects were generated. Due to small sample size, 95 % bootstrap confidence intervals based on 10,000 bootstrap samples were obtained.

## Results

FNS diagnoses were confirmed by prominent somatoform dissociative symptoms: SDQ-20 scores significantly distinguished FNS and HC, and within the FNS sample, SDQ-20 scores distinguished patients with multiple dissociative movement and sensitivity disorders from patients with either single movement or sensitivity disorder (see Table 2). FNS patients received more comorbid PTSD diagnoses (24 %) than HC (*χ*^2^ (1) = 12.84, *p* < 0.001), and FNS patients with multiple dissociative movement and sensitivity disorders were more frequently diagnosed with comorbid PTSD (33 %) than patients with either movement or sensitivity disorders (7 %, *χ*^2^ (1) = 3.85, *p* = 0.05). FNS patients reported higher psychological strain (SCL90R-GSI) than HC (*t*(88) = 8.56, *p* < 0.001), and patients with multiple movement and sensitivity disorders reported higher psychological strain than patients with either movement or sensitivity disorder (*U* = 121, z = −2.5, *p* < 0.05). Across participants, SDQ-20 scores and SCL90R-GSI correlated with *r* = 0.75, *p* < 0.001.

**Table 2:** Functional neurological symptoms and experience scores per group and inferential statistics on group comparisons

	FNS patients (*n* = 45)		Healthy comparison subjects (*n* = 45)		FNS patients vs. HC	F44.4/F44.6 vs. F44.7
	*M* ± SD	median *(range)*	*M* ± SD	median *(range)*		
**SDQ-20**	33.3±9.5	31 *(27–38)*	21.4±1.7	21 *(20–22)*	*t*(88) *=* 8.63***, *d* = 0.68	*U* = 117.5, *z* = -2.59*, *r* = -0.36
**ETI**						
Group					*F*(1, 88) = 12.59***, η^*2*^ = 0.13	
Domainc					*F*(1.1,97) = 31.4***, η^*2*^ = 0.26	
Group x domain					*F*(1.1,97) = 13.19***, η^*2*^ = 0.13	
*Group simple effects by ETI domain*						
General	2.5±3.7	1.17 *(0.43–2.93)*	1±1.6	0.3 *(0.09–1.48)*	*U* = 625.5, z = -3.13**, *r* = -0.33	*n. s.*
Physical	4.1±6.4	1.44 *(0.11–4.15)*	2.6±3.2	1.11 *(0.11–3.17)*	*n. s.*	*n. s.*
Emotional	20.8±27.5	10 *(1.25–34.19)*	4.9±10.6	0.75 *(0–5.19)*	*U* = 539, z = -3.89***, *r* = -0.41	*n. s.*
Sexual abuse	0.6±2	0 *(0–0)*	0.1±0.2	0 *(0–0.03)*	*n. s.*	*U* = 150, z = -2.48*, *r* = -0.34
**LEQ**						
Group					*F*(1, 88) = 10.91**, η^*2*^ = 0.11	
Group x domain					*F*(1, 88) = 58.12***, η^*2*^ = 0.4	
*Group simple effects by LEQ domain*						
sum-	21.3±12.5	23 *(10.5–31.5)*	6±5.1	4 *(2–8)*	*F*(1,88) = 57.64***, η^*2*^ = 0.4	*U* = 138.5, z = -2.08*, *r* = -0.37
sum+	10.9±9.2	9 *(2.5–16)*	14.9±11.8	10 *(6–24)*	*n. s.*	*n. s.*
**ERQ**_**S**_	4.1±1.6	4.25 *(3–5.25)*	3.1±1.3	2.75 *(2–4.13)*	*t*(88) = 3.18**, *d* = 0.32	*n. s.*
**TAS-26**	2.6±0.6	2.61 *(2.28–2.89)*	2±0.4	1.94 *(1.67–2.22)*	*t*(88) = 6.61***, *d* = 0.58	*n. s.*

*Note.* FNS = functional neurological symptoms; HC = healthy comparison subjects; F44.4/F44.6 = dissociative movement or sensitivity disorder; F44.7 = multiple dissociative movement and sensitivity disorder; SDQ-20 = FNS symptom severity verified using the *Somatoform Dissociation Questionnaire*; ETI = *Early Trauma Interview* including the domains: general traumata, emotional neglect/abuse, physical neglect/abuse and sexual abuse; LEQ = *Life Events Questionnaire* with: LEQ sum- = recent negative life events; LEQ sum+ = recent positive life events; ERQ_s_ = suppressive emotion regulation style assessed using the *Emotion Regulation Questionnaire*; TAS-26 = *Toronto Alexithymia Scale*. ***: *p* < 0.001, **: *p* < 0.01, * *p* < 0.05, *n. s.*: not significant, p > 0.05

### Stressful experiences:

**Fig. 1:**
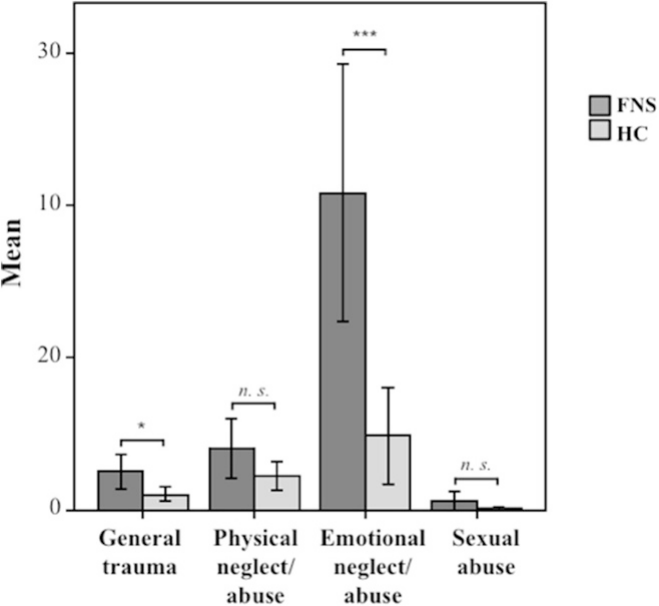
Adverse Childhood Experiences. Bar graph illustrating the sum scores (events x frequency) per ETI domain (general traumata, physical neglect/abuse, emotional neglect/abuse, sexual abuse) and group (FNS, HC). Asterisks indicate significance of * *p* ≤ 0.05, ** *p* < 0.01, *** *p* < 0.001

FNS patients reported more ACE than the HC group (Table 2). An interaction group x ACE domain confirmed more emotional neglect/abuse and more general traumata in FNS patients than in the HC group, while physical abuse and sexual traumata did not differ between groups (Fig. 1). FNS severity (per SDQ-20 score) varied with emotional stress (*r* = 0.41, *p* < 0.001) and general traumata (*r* = 0.39, *p* < 0.001), while patients with multiple movement and sensitivity disorders reported more childhood sexual abuse than patients with either a movement or a sensitivity disorder (Table 2). Groups (FNS patients vs. HC) also differed in the experience of recent negative LE, whereas positive life events did not differ. FNS severity varied with recent negative life events (LE; *r* = 0.59, *p* < 0.001). FNS patients with multiple movement and sensitivity disorders reported more negative LE than patients with either a movement or a sensitivity disorder.

### Emotion processing

Patients reported higher alexithymia (TAS-26) and more suppressive emotion regulation style than HC (ERQs; Table 2). The severity of FNS varied with alexithymia (*r* = 0.49, *p* < 0.001) and suppressive emotion regulation style (*r* = 0.3, *p* < 0.05).

The multiple regression analysis including ACE, LE and measures of emotion processing indicated additive effects of negative LE (β_LEQ-_ = 0.37, *p* < 0.01), emotional ACE (β_ETI_ = 0.21, *p* < 0.05) and alexithymia (β_TAS-26_ = 0.28, *p* < 0.05) on FNS severity, with no additional variance explained by a suppressive emotion regulation style (ERQ_S;_ adjusted *R*^*2*^ = 0.4; *p* < 0.01).

### Mediation analysis

**Fig. 2:**
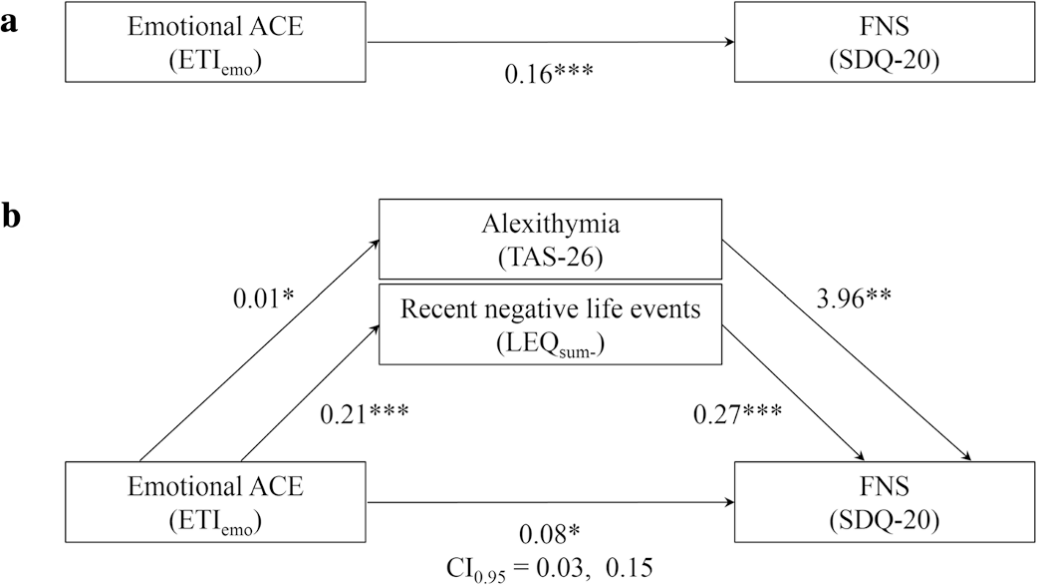
Path analyses showing the relationship between emotional ACE, FNS severity, recent negative life events and alexithymia. **a** Association between emotional ACE (per ETI score), and FNS severity (per SDQ-20 score). **b** Association between emotional ACE and FNS severity through recent negative life events (per LEQ_sum-_) and alexithymia (per TAS-26). Unstandardized beta coefficients are shown for each path. Asterisks indicate significance of * *p* ≤ 0.05, ** *p* < 0.01, *** *p* < 0.001

Only the significant predictors of FNS severity – emotional ACE (ETI_emo_), negative LE (LEQ_−_) and alexithymia (TAS-26) – were included in the mediation analyses. The positive total effect of emotional ACE on FNS severity (Fig. 2a) as well as positive direct effects of alexithymia and negative LE on FNS severity (Fig. 2b) were confirmed. While the direct effect of emotional ACE on FNS severity remained significant after adjusting for alexithymia and negative LE (Fig. 2b), a bootstrapping procedure revealed a positive total indirect effect of emotional ACE on FNS through negative LE and alexithymia (bias-corrected CI_0.95_ = 0.03, 0.15). These results indicate that the relationship emotional ACE-FNS is partially mediated by level negative LE and alexithymia.

## Discussion

“That pulled the rug out from under my feet!” – the present data demonstrate a significant impact of stress load early in life, which accumulates in adulthood in FNS patients. In particular, *emotional* adversities and alexithymia influence the severity of FNS. The data also indicate a mediating role of alexithymia in the relationship between ACE and FNS severity. These results should shape the modeling of FNS as a ‘conversion’ of emotional responses to stressful experiences into bodily FNS.

The more detailed description of the types and times of stressful experiences in regard to their impact on FNS goes beyond previous reports that demonstrated the impact of traumatic experiences on FNS (e.g., [9, 3, 4, 11–13]). The present results draw particular attention to the (often neglected) harmful effects of emotional experiences, including parental neglect, verbal aggression, devaluation and humiliation within families and by peers. FNS severity (in single as well as multiple sensory and/or motor domains) was evidently related to emotional neglect and abuse. The present results also validate the impact of sexual abuse on the development of conversion disorder [24, 11, 3]. In the present sample, patients with multiple FNS more frequently reported sexual abuse, whereas patients with single-domain FNS showed less sexual abuse. As a consequence of the different frequencies, sexual abuse did not differ significantly between the entire patient sample and HC when averaged across FNS patients. Since patients with multiple FNS showed more psychological strain and reported more ACE in most domains (including sexual abuse), conversion disorder (F44.7) can be considered the most severe disorder of those under investigation here.

The present results emphasize an impact of accumulating stress load, showing a relationship between recent negative life events and FNS severity. While the latter relationship has been reported before [11], the present results draw attention to a potential interaction between both ACE and negative LE in their impact on FNS. This relationship may indeed signal a dose effect, in that the amount of stress fosters symptom or illness severity: higher emotional ACE and negative LE load varied with higher FNS severity, comorbid PTSD and overall psychological strain. A dose effect of accumulating stress on disorder severity has been suggested for severe mental disorders such as PTSD [54], major depressive disorder, borderline personality disorder [55–57], and dissociative identity disorder [16]. Individuals who suffered from early trauma later suffered from physical and mental disorders [26, 58, 59] or conversion disorder [11].

The present contribution of LE as a mediator of ACE effects on FNS may also signal a sensitizing effect, in that stress during childhood fosters vulnerability. A sensitizing role is conceivable in the conceptual framework of sensitive periods of brain and neuroendocrine systems maturation, during which emotional and sexual traumata exert particularly harmful influences, thereby sensitizing for psychopathological development [20, 23].

In the conceptual framework of FNS as conversion disorder or somatoform dissociation, emotional ACE can thus indeed be assumed to influence FNS by means of altered emotion processing. Conversion models describe the redirection of emotion expression in bodily symptoms upon trauma [60, 61]. Altered emotion processing manifest in alexithymia has been suggested to facilitate somatoform symptoms, dissociation and FNS [4, 6, 28, 62]. Therefore, FNS might be conceived of as such a dysfunctional bodily expression of dysfunctional emotion processing. Based on a reasonable sample of heterogeneous FNS, the present empirical results on the relationships between ACE and alexithymia as well as on alexithymia and FNS are in line with this (still rather theoretical) model, although the direct influences or mediating roles of altered emotion processing remain to be further substantiated.

The conclusions are constrained by further limitations: The experience of ACE, including the prominence of emotional ACE and stress load across life is not specific to FNS, but has been reported for a number of severe mental disorders (e.g., [56]). This could emphasize the – perhaps often underestimated – significant impact of emotional experiences on the development of psychopathology in general. The extent to which emotional adversities are even more influential in mental disorders with emotional involvement, as discussed for conversion disorders and FNS, remains to be clarified by a direct comparison between diagnostic groups.

The present assessment focused on two aspects of emotion processing, alexithymia and emotion regulation style. While both were related to FNS severity and to emotional ACE, only alexithymia met the criteria for further mediation analyses and proved to partially mediate the relationship between ACE and FNS. Other aspects of emotion processing need to be evaluated as factors and mediators in FNS development before a conclusion on the specific role of alexithymia can be justified. Moreover, cognitive functions such as attention and memory have to be considered as mediators between ACE and FNS [60, 61]. As an example, Schauer and Elbert [17] proposed shut-down dissociation as a consequence of extreme helplessness in traumatic situations. If this way of coping with stress and trauma is reinforced by further threat and consolidated by subsequent avoidance, PTSD becomes likely (see also [19]). Whether and to what extent such dissociative processes may have affected FNS in those patients who reported the highest stress load (including emotional and sexual abuse) and were diagnosed with comorbid PTSD cannot be specified without detailed individual histories.

Moreover, validation of a conversion or dissociation model of FNS requires the consideration of further factors that contribute to the potentially multifactorial FNS genesis. These include, for instance, the integration of bodily symptoms and longitudinal studies with repeated assessments to monitor the development of chronic FNS.

Finally, the reliability of retrospective self-reported data on childhood experiences is always a matter of concern and may constrain the validity of results. Standardized instruments with adequate psychometric properties [41, 44] are available for the assessment of ACE and produce replicable results across many studies. The present study used such instruments despite the authors’ awareness of their remaining limitations.

## Conclusions

The present results lend support to the notion that adverse childhood experiences contribute to FNS severity and accentuate the impact of emotional neglect and abuse. In addition, the present data support the assumption that emotional adversities alter emotion processing, which both in turn influence FNS severity. These results advance the understanding of FNS and should inform modeling of FNS as a trauma-induced ‘conversion’ of emotional stress responses into bodily symptoms. Although the present mediating analyses are still insufficient for clarifying the complex interaction, the impact of emotional adverse experiences and of alexithymia in FNS should be considered in diagnostic and therapeutic procedures. Diagnostic assessment for treatment assignment should include the assessment of the history of emotional and traumatic stress and the ability to identify emotional responses related to those experiences and their potential (acquired) association with bodily responses in the traumatic situation. Intervention should focus on this dysfunctional association and train emotion processing skills.

## Additional file

Additional file 1: **Detailed Description of Instruments (Psychometric Characteristics).** Additional information on the psychometric characteristics of each (German version) of the self-report instrument used.

## Abbreviations

ACE: Adverse childhood experiences
DSM: Diagnostic and statistical manual of mental disorders
ERQ: Emotion regulation questionnaire
ERQs: (Sum score of) suppressive emotion regulation measured with ERQ
ETI: Early trauma inventory
FNS: Functional neurological symptoms
F44.4: ICD-10 code for dissociative motor disorders/dissociative movement disorder
F44.5: ICD-10 code for dissociative convulsions
F44.6: ICD-10 code for dissociative anaesthesia and sensory loss/dissociative sensitivity disorder
F44.7: ICD-10 code for mixed dissociative [conversion] disorder
ICD-10: International statistical classification of diseases and related health problems, 10th revision
LE: Recent negative life events
LEQ: Life events questionnaire
LEQsum: Sum score of recent negative life events reported in life events questionnaire
PTSD: Post-traumatic stress disorder
SDQ-20: Somatoform dissociation questionnaire
SCL90R: Symptom checklist 90 revised
SCL90R-GSI: Sum score for experienced psychological strain in SCL90R (GSI: “global severity index”)
TAS-26: Toronto alexithymia scale 26

## Footnotes

1 FNS is used in DSM-5 as descriptive term without addressing hypothetical mechanisms. The additionally used terms *dissociative disorder* or *dissociative symptoms* refer to the (ICD-10-)diagnoses and their emphasis on dissociation as central to the disorder. Furthermore, we try to focus on somatoform dissociative symptoms, i.e. physical deficits such as hypesthesia or gait disturbance, thereby acknowledging the often neglected contrast to psychoform dissociative symptoms such as amnesia or identity disorders; for a definition see [8, 16].

2 Regarding FNS in the present sample, ICD-10 and DSM diagnostic criteria overlap, as FNS (DSM-5) include negative somatoform dissociative/conversion symptoms as used in ICD-10 and DSM-IV.

3 Internal consistency (Cronbach’s α = 0.92) and test-retest reliability (*r*_*tt*_ = 0.89) reported for German version. Construct and criterion validity were confirmed by [37].

4 Test-retest reliability confirmed by correlation coefficients ranging from *r*_*tt*_ = 0.68 to *r*_*tt*_ = 0.80 [39]. Internal consistency of the GSI : α = 0.97 [61].

5 Internal consistency (Cronbach’s α = 0.68), good convergent validity [63].

6 Internal consistency: α = 0.88; good convergent validity (*r* > 0.72 [44]).

7 The individual onset of puberty was defined as the age at menarche (women) and as the age at voice break or the age at onset of shaving (men), respectively.

8 Test-retest reliability: *r*_*tt*_ = 0.78 to *r*_*tt*_ = 0.83 [46]. Construct validity has been shown by [45].

9 Internal consistencies α = 0.67 to α = 0.84; good convergent validity [49].

10 Internal consistencies: Cronbach’s α = 0.76 for cognitive reappraisal and α = 0.74 for suppression; good convergent validity [50].
