# Supplementary material for: “That pulled the rug out from under my feet!” – adverse experiences and altered emotion processing in patients with functional neurological symptoms compared to healthy comparison subjects
Source: BMC Psychiatry. 2015 Jun 24;15:133. doi: 10.1186/s12888-015-0514-x (PMC4477601; doi:10.1186/s12888-015-0514-x)
Supplement: Additional file 1: — Detailed Description of Instruments (Psychometric Characteristics). Additional information on the psychometric characteristics of each (German version) of the self-report instrument used. [file 12888_2015_514_MOESM1_ESM.docx]

**Additional File 1: Detailed Description of Instruments (Psychometric Characteristics)**

*Somatoform Dissociation Questionnaire*

Functional neurological symptom (FNS) severity was verified with the Somatoform Dissociation Questionnaire [9,37]. The SDQ-20 assesses the frequency (percentage) of somatoform dissociation experienced during the preceding 12 months with 20 items, each evaluated on a 5-point Likert-scale ranging from (1) ‘this applies to me not at all’ to (5) ‘this applies to me extremely’. Higher scores between a minimum score of 20 and a maximum score of 100 indicate more somatoform dissociation. The German version displays high reliability, internal consistency (Cronbach’s α = 0.92) and test-retest reliability (*r_tt_* = 0.89). Construct and criterion validity were confirmed [37].

*Posttraumatic Stress Scale - Interview*

Comorbid PTSD was diagnosed with the Posttraumatic Stress Scale – Interview [40,41], Based on DSM-IV diagnosis criteria, meeting criteria A-F was prerequisite for a PTSD diagnosis. Three subscales assessed the frequency of symptoms shown in the previous four weeks on a 4-point Likert-scale from (0) ‘not at all/once in the previous month’ up to (3) ‘five or more times per week/almost every day’: reliving (B criterion: one out of five symptoms), avoidance (C criterion: three out of seven symptoms), and hyper-arousal (D criterion: two out of five symptoms). PSSI shows good internal consistency (Cronbach’s α = 0.68) and convergent validity [64].

*Symptom Checklist-90-R*

The global severity index (GSI) from the Symptom Checklist-90-R [38] represents the sum of symptoms on the dimensions of somatization, obsessive compulsivity, interpersonal sensitivity, depression, anxiety, hostility, phobic anxiety, paranoid ideation, psychoticism the subjectively experienced distress, each evaluated on a 5-point Likert-scale from (0) ‚not at all’ to (4) ‚extreme’. Test-retest reliability is considered adequate; correlation coefficients ranging from *r_tt_* = 0.68 to *r_tt_* = 0.80 [39]. Internal consistency of the GSI is high with α = 0.97 [61].

*Early Trauma Inventory*

Childhood adverse experiences were screened with the German version of the Early Trauma Inventory [42,43]. The ETI assesses the amount (number, frequency, and severity) of childhood adversities in the four dimensions ‘general traumata’, ‘emotional abuse/neglect’, ‘physical abuse/neglect’ and ‘sexual abuse’. For each reported event the age when it started and the age when it terminated are specified, and the event frequency within each domain and year is encoded on a 7-point Likert-scale ranging from (0) ‘never within this year’ to (6) ‘several times a day’. These frequencies were summed up for each year of experience, and the latter frequencies were summed up for the time period before the individual onset of puberty constituting the adversity score for comparison between subjects and in relation to symptom scores. Statistical analyses was based on the mean per domain. The psychometric criteria are reported as satisfactory [42] with good convergent validity (correlations > 0.72) and internal consistency of α = 0.88 [44].

*Life Events Questionnaire*

Recent negative and positive life events were screened with the Life Events Questionnaire [46,45]. Participants were asked whether they had experienced a certain event in the preceding twelve months and rated the subjectively experienced effect of this event on a 3-point Likert-scale. The correlation coefficients of the test-retest reliability range from *r_tt_* = 0.78 to *r_tt_* = 0.83 [46]. Construct validity has been shown [47].

*Toronto Alexithymia Scale*

The Toronto Alexithymia Scale [49,47-48] evaluates alexithymia by 26 items on three scales (‘difficulties to identify feelings’, ‘difficulties to describe feelings’ and ‘external oriented thinking’), which add up to a total score. Each item was assessed on a 5-point Likert-scale from (1) ‘strongly disagree’ to (5) ‘strongly agree’. Internal consistencies reach from α = 0.67 to α = 0.84, convergent validity has been demonstrated [49].

*Emotion regulation questionnaire*

Habitual emotion regulation strategies were assessed with the Emotion regulation questionnaire [31,50]. Items cover the regulation strategies ‘cognitive reappraisal’ (six items) and ‘suppression’ (four items), each evaluated on a 7-point Likert-scale from (1) ‘strongly disagree’ to (5) ‘strongly agree’. Both scales show good reliability for (Cronbach’s α = 0.76 for ‘cognitive reappraisal’ and α = 0.74 for ‘suppression’), and convergent validity [50].

**Additional References**

1. Griesel D, Wessa M, Flor H: Psychometric properties of the German version of the Posttraumatic Diagnostic Scale (PDS). Psychological Assessment 2006, 18:262–268
